# Annotations

**Published:** 1909-11-20

**Authors:** 


### Annotations.

### Women and the R.C.S.

A further step in the women's movement was won last week, when the meeting of the Council of the Royal College of Surgeons on November 11 announced that the by-laws relating to the admission of women had been signed by the Home Secretary, the Lord Chancellor, and the Lord Chief Justice. The privileges attaching to the diploma will entitle women to compete for the Collegiate and Jacksonian prizes, and will give them admission by right and not merely by courtesy to the museum, the library, and to all lectures. It has taken time to surmount the conservative opinions of medical men and men generally in this matter, but the success of this attempt to gain equality of status for both sexes in the College is interesting, as it means that, as far as medicine is concerned, little remains to be done before equal facilities are offered on every point to women practitioners. It is curious that medicine, which was held at first a profession peculiarly unsuited to women, should be one of the first to recognise her claim. We imagine that law, the genius of which is its conservatism, and the idol of which is the awful power of precedent, will offer them much greater opposition. But when the Law Society has followed the example of the R.C.S., then perhaps we may hope that even Cambridge and Oxford will acknowledge, by the conferring of degrees, the equality they have already extended to both sexes in fact.

### Errors in Diagnosis.

It is a curious illustration of the vagaries of human nature and human thought that the doctor is one of the few beings who may never admit error. Medical men, in fact, are almost compelled by the mental attitude of their patients to assume an infallibility pontifical in its completeness. Though the public is only too ready to proclaim aloud that its ailment has been misdiagnosed or mistreated, usually without the slightest foundation in fact, yet it does not permit the practitioner to admit a mistake and retrieve it. This trait, no doubt, accounts for the rarity with which medical men discuss their own mistakes and errors, though there are some who are not equally chary of dilating on those of their colleagues or neighbours. On infrequent occasions some outspoken individual does lay bare his soul in this manner, however, and as a rule the narration is full of interest and instruction. As a rule, this is done only by those whose method, experience, knowledge, and skill are distinctly out of the ordinary, so that the mistakes they mention are merely examples of the old truth that medicine is not, and never can be, an exact science in the sense that mathematics is. At the October meeting of the South-West London Medical Society Dr. M. Mackintosh related a series of personal experiences, including several of mistakes in diagnosis, which are most valuable examples of the pitfalls that surround the footsteps of the general practitioner, and of the way in which the wariest may be entrapped. This form of confession is one to be distinctly encouraged, for it is hard to imagine any professional paper for a local medical society more calculated to be useful to fellow-members, old or young. At the same time, the publication of such experiences in any but the medical Press would be most disastrous, simply for the reason mentioned: that the public is not yet sufficiently educated up to the conception that there are errors which are not merely pardonable, but unavoidable. We congratulate the author of the paper above mentioned on his honesty, and feel sure that similar papers by practitioners of standing are of the greatest value and encouragement to younger men.

### American Football.

American football has been long noted for its brutality, but it was hoped that the new rules which were largely improved by the efforts of Dr. Elliot, late President of Harvard, would have mitigated the dangers of the game. It would seem that this is not the case, for in the first few weeks of the present season two broken necks have occurred, one to a player in the Naval College team, and another to a member of the Army College team at West Point. The authority of the public services may perhaps now succeed in moderating the severity of the game where the private initiative of the universities has failed. The dangers of football as played on the "campus" of American schools are not accidental, but inhere in the nature of the game. It would seem that medical men could do something to put an end to the present manner of playing football. But this supposition is contradicted by the actual state of affairs, for many of the athletic directors in the universities are medical men. As they are judged by the success of their team, it is they who encourage the very types of play which are so dangerous to life and limb. During the present season, scarcely a month old, 14 deaths are already reported from accidents during football; and yet the sporting writers are endeavouring to belittle the connection of the game with these, alleging that a man might break his neck (as has happened) in falling over a tennis-net, or contract septicæmia after a scratch from a nail or piece of glass. It would seem obvious enough that the advantages of manly training given by football might be secured without its present grave danger by modifying or reforming the American game in the direction of the present Rugby game as played in Greater Britain. All players of the English game can affirm its rigorousness and use as a developer of resourcefulness, grit, and stamina. Moreover, both experience and statistics show its relative lack of serious danger even to limb. American progressiveness seems to be asleep here, and the common boast that they are not bound by tradition and can assimilate the best which other countries afford seems to fail the Americans, at least where its football problem is concerned.

